# Environmental Enrichment Rescues Visually-Mediated Behavior in Ten-m3 Knockout Mice During an Early Critical Period

**DOI:** 10.3389/fnbeh.2020.00022

**Published:** 2020-02-25

**Authors:** James Blok, Dylan A. Black, Justin Petersen, Atomu Sawatari, Catherine A. Leamey

**Affiliations:** Department of Physiology, School of Medical Sciences and Bosch Institute, Faculty of Medicine and Health, University of Sydney, Camperdown, NSW, Australia

**Keywords:** visual development, plasticity, environmental enrichment, ipsilateral, subcortical, ten-m/Teneurin/Odz, looming stimulus

## Abstract

Environmental enrichment (EE) has been shown to promote neural plasticity. Its capacity to induce functional repair in models which exhibit profound sensory deficits due to aberrant axonal guidance has not been well-characterized. Ten-m3 knockout (KO) mice exhibit a highly-stereotyped miswiring of ipsilateral retinogeniculate axons and associated profound deficits in binocularly-mediated visual behavior. We determined whether, and when, EE can drive functional recovery by analyzing Ten-m3 KO and wildtype (WT) mice that were enriched for 6 weeks from adulthood, weaning or birth in comparison to standard-housed controls. EE initiated from birth, but not later, rescued the response of Ten-m3 KOs to the “looming” stimulus (expanding disc in dorsal visual field), suggesting improved visual function. EE can thus induce recovery of visual behavior, but only during an early developmentally-restricted time-window.

## Introduction

Environmental enrichment (EE) has been reported to confer numerous benefits to neural function. In comparison to a standard laboratory housing environment (SE), EE typically provides access to larger cages and social groups, running wheels and toys that enhance the multisensory experience. EE has been shown to accelerate development, enhance neural plasticity, as well as having beneficial effects on models of neurodegenerative disease (Nithianantharajah and Hannan, 2006), and neurodevelopmental disorders (Kondo et al., 2008, 2016; McOmish et al., 2008; Begenisic et al., 2015).

In the visual cortex, EE has been shown to extend the usual juvenile period of cortical plasticity which enables recovery from amblyopia into adulthood (Sale et al., 2007; Baroncelli et al., 2010, 2016; Scali et al., 2012; Greifzu et al., 2016), as well as accelerating the maturation of neural circuits in young mice (Cancedda et al., 2004; Ciucci et al., 2007). Benefits of EE on hippocampal function have also been demonstrated (Bernstein, 1973; van Praag et al., 2000; Speisman et al., 2013). Although highly beneficial, an understanding of where and how EE acts at a circuit level is lacking. In particular, the capacity for EE to induce functional repair of miswired circuits has not been well-characterized. Increased understanding of this may have important implications for the development of therapies for neurodevelopmental disorders which are characterized by aberrant neural connectivity.

The early visual pathway of Ten-m3 knockout (KO) mice provides an excellent model to explore this issue. Ten-m3 belongs to a family of type II transmembrane proteins which have been shown to regulate a number of developmental processes such axonal guidance, synapse formation and dendritic structure (Leamey et al., 2007; Young and Leamey, 2009; Dharmaratne et al., 2012; Hong et al., 2012; Mosca et al., 2012; Antinucci et al., 2013, 2016; Young et al., 2013; Glendining et al., 2017; Berns et al., 2018; Leamey and Sawatari, 2019). Most notably for this project, the phenotype of Ten-m3 KO mice is characterized by a highly stereotyped miswiring of ipsilateral retinal projections within the dorsal lateral geniculate nucleus (dLGN; Leamey et al., 2007). Unlike wildtype (WT) mice, where ipsilateral projections consistently map to the dorsomedial region of the dLGN, in standard-housed (SE) Ten-m3 KOs ipsilateral axons terminate in an elongated strip that extends far into ventrolateral dLGN (Leamey et al., 2007). The mapping deficits are transferred to the primary visual cortex (V1; Merlin et al., 2013). This results in misalignment of ocular inputs to V1 and is associated with profound functional deficits: SE Ten-m3 KO mice are unable to perform behavioral tasks which engage patterned binocular vision (Leamey et al., 2007). Interestingly, acute monocular inactivation was shown to restore visual function suggesting that inappropriate interactions between inputs arising from the two eyes cause the visual deficits (Leamey et al., 2007; Merlin et al., 2013).

We have recently shown that 6 weeks of EE from birth, but not from weaning or later, is able to induce a significant pruning of mismapped ipsilateral retinogeniculate terminals in Ten-m3 KOs (Eggins et al., 2019). Of note, the most aberrant projections showed the greatest retraction in enriched KO mice. We sought to determine whether these changes were associated with the recovery of binocularly-mediated visual behavior in enriched Ten-m3 KO mice. We assessed the response of WT and Ten-m3 KO mice to a dorsally-presented rapidly expanding disc. This “looming” stimulus simulates the approach of an aerial predator and drives an innate defensive response to seek shelter (Yilmaz and Meister, 2013). Mice that had experienced EE from birth, weaning or adulthood were compared to age-matched SE controls. Unlike WTs, SE Ten-m3 KOs from all age groups responded poorly to the stimulus. Exposure to EE during adulthood or from weaning did not rescue behavior in KOs, but those enriched from birth displayed a significant recovery. We conclude that EE from birth is able to rescue ethologically-appropriate visually-mediated behavior.

## Materials and Methods

This study was carried out in accordance with the recommendations of the Australian Code for the care and use of animals for scientific purposes (Edition 8), National Health and Medical Research Council Guidelines, Animal Welfare Committee. The protocol was approved by the Animal Ethics Committee of the University of Sydney.

All animals were housed in climate-controlled rooms (~23.5°C, 40–70% humidity) at the University of Sydney Animal Housing Facility on a fixed 12/12 h light/dark cycle. Standard mouse chow and water were provided *ad libitum*.

### Animals

Ten-m3 KO and WT mice (described in Leamey et al., 2007) were obtained by breeding female heterozygotes with male heterozygotes in standard cages (see below). Mice were genotyped using tissue biopsy followed by polymerase chain reaction, as described previously (Leamey et al., 2007). A total of 121 mice were used in this study, with 9–12 mice in each of the 12 groups analyzed (2 genotypes × 2 housing conditions × 3 ages).

### Standard and Enriched Housing

Animals raised in standard conditions were housed in individually ventilated plastic cages (32.5 cm × 15 cm × 16.5 cm). Each cage housed 2–5 mice and contained shredded paper for nesting, an igloo, food hopper, and water bottle.

Animals exposed to in EE were housed in large, 2-storey cages (45 cm × 37.5 cm × 39 cm). Each cage housed 3–10 mice and contained a mouse igloo with running wheel, one long and one short toilet paper roll, half a tissue box, 3–5 marbles, 1–2 ping pong balls, multi-colored paddle pop sticks tied together with multi-colored pipe cleaners, two high contrast visual stimuli (a checkerboard and a diagonal grating), and three scented plush ball toys. These objects were chosen in order to stimulate as many senses as possible; the running wheel provided access to voluntary exercise. The positions of enrichment objects in the EE cages were changed three times a week for added stimulation and replaced/re-scented as required.

Dams were either transferred to individual standard housing cages (1 dam per cage), or in the case of EE from environmental enrichment from birth (EE-B) in pairs into enrichment cages (2 dams per cage), in the last 2–3 days of pregnancy. Pups were weaned into sex-specific cages at 3 weeks of age (postnatal day 21). Pups allocated to commence EE from environmental enrichment from weaning (EE-W) were weaned from dams housed in standard conditions into EE cages. Mice allocated to commence EE in environmental enrichment from adulthood (EE-A) were transferred into sex-specific enrichment cages at 3–6 months of age. Mice from all three enrichment groups experienced EE conditions for 6 weeks prior to behavioral testing (age of assessment: 6 weeks for EE-B, 9 weeks for EE-W, and 5–8 months for the EE-A groups).

For SE age-matched control animals, mice were bred in conventional cages. They were weaned at 3 weeks into sex-specific standard cages and raised until they reached the appropriate age for assessment [6 weeks for standard environment control for birth group (SE-B), 9 weeks for standard environment control for weaning group (SE-W), and 5–8 months for standard environment control for adult group (SE-A) groups].

### Behavioral Testing

Behavioral testing was conducted in a custom-made, open-top glass aquarium (48 cm × 48 cm × 30 cm). All four walls were covered with matte-black Perspex to minimize reflection of the stimulus. A clear red Perspex sheet was placed under the base of the arena to allow for the recording of behavioral responses using a video camera (Microsoft, WA, USA) placed underneath the test chamber. The filtered red light was used for ambient illumination within the testing room.

The testing arena featured a shelter in one corner (12.5 cm × 10.5 cm × 7.5 cm) and a small round dish (6 cm in diameter) placed in the center of the open field enclosure. These features were present throughout the habituation and testing phases of the experiment. Stimuli were presented *via* a liquid crystal display monitor placed face-down on top of the aquarium.

The “looming” stimulus (generated using open-access software (PsychoPy, Jonathan Peirce, University of Nottingham) consisted of an expanding black circle/disk (2–20° of visual art at a rate of 72°/s) presented against a gray background (Yilmaz and Meister, 2013). The stimulus was held for 250 ms and repeated 15 times with 500 ms interstimulus intervals. Subsequently, the screen went black and the trial was terminated if the mouse had not already escaped. The center of expansion was situated directly over the central circular dish.

On the day prior to testing, mice were individually habituated in the test chamber for 10 min. A sunflower seed was placed in a centrally located dish to encourage exploration. The monitor was programmed to display a uniform gray screen during this period.

Following habituation, mice were returned to their home cages and food-deprived overnight. This step was included to further motivate the mice to approach the dish placed in the center of the open field. On the subsequent day of testing, mice were placed once again in the aquarium. Subjects were permitted to freely explore the arena while being monitored *via* live video feed from the camera placed beneath the chamber. After 2 min, a single behavioral trial was initiated. If during this period, the tip of the subject’s nose crossed the circumference of the circular dish, the looming stimulus was activated manually, and subsequent behavior of the animal was recorded. If the subject did not approach the center of the arena within 10 min the subject was returned to its home cage.

### Behavioral Analysis

Movement of individual mice (based on the position of their nose-tip) within the testing chamber was tracked frame by frame at 167 ms intervals using the MTrackJ plugin from ImageJ (NIH). The trajectories of each mouse were plotted graphically, super-imposed for each group (age * housing * genotype) and used for qualitative analysis.

Movement was tracked from the onset of the stimulus to when the nose of the subject first crossed boundaries of three pre-determined “escape areas” (a region that extends along the length of one wall of the arena in line with the opening of the shelter designated as the “bottom area” (solid lines on the bottom of trajectory panels depicted in Figure 2); a region extending along the entire length of the opposite wall to the “bottom area,” and of comparable distance from the arena center referred to as the “top area” (top solid lines depicted in Figure 2); and finally the shelter itself (“S” in trajectory panels; Figure 2), or until the end of stimulus presentation (approximately 20 s).

**Figure 1:**
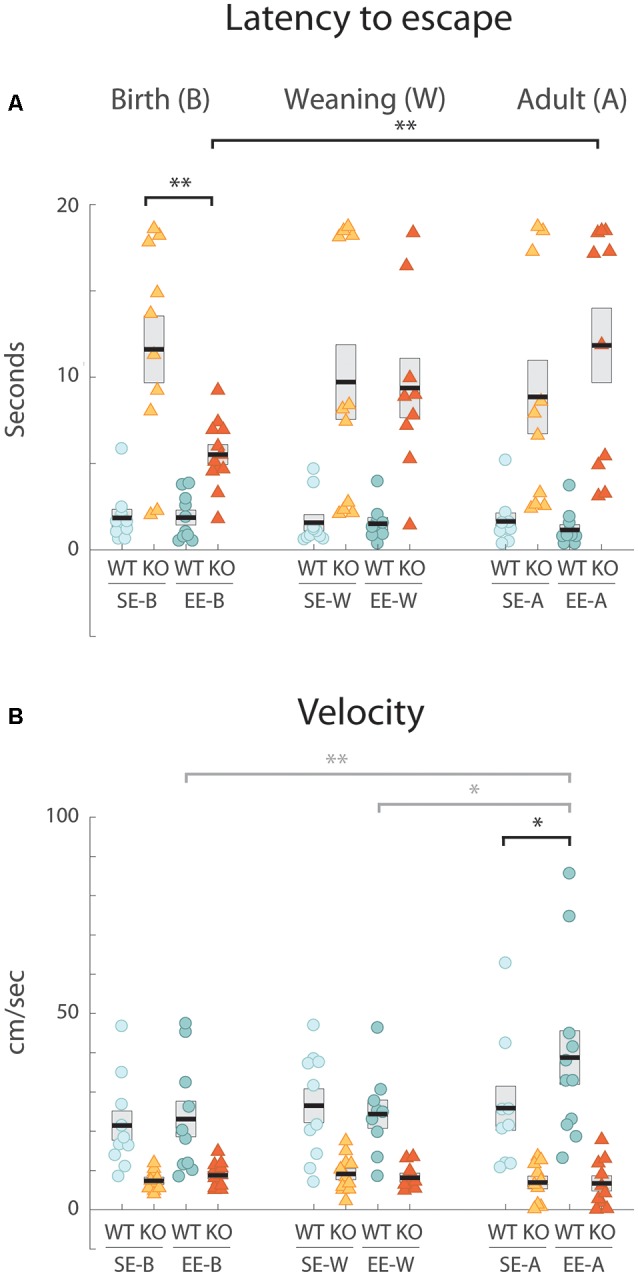
Environmental enrichment (EE) from birth improves a defective response to the looming stimulus exhibited by standard-housed Ten-m3 knockout (KO) mice. Data derived from individual animals in each group is shown. Thick black lines show group means, boxes indicate SEM. Housing groups are separated by age with the birth cohort shown on the left, weaning groups in the middle, and adults on the right. The same shading and color coding for housing [lighter shade for standard-housing groups (SE); darker for all EE groups] and genotype [blue for wildtype (WT); red for KO] is used across age groups for ease of comparison. All SE WTs are shown in light blue and all EE WTs are shown in darker blue. Accordingly, all SE KOs are shown in orange and all EE KOs are shown in red. **(A)** Latency in seconds from initiation of looming stimulus presentation to reaching escape zone. WTs consistently exited the maze quickly, with mean values for all age and housing groups under 2 s. All SE KO cohorts had much more variable latencies which were significantly longer than for all SE WTs (*p* < 0.001; not marked). EE from birth induced a significantly reduced latency in KOs environmental enrichment from birth [(EE-B) KO compared to SE-B-KOs: **p = 0.001]. EE-B KOs were not significantly different from EE-B WTs (*p* = 0.053). No decrease in latency was seen for KO groups with EE from weaning or in adulthood compared to SE KO controls. These groups were also significantly different from their respective WT groups (*p* < 0.001; not marked). While no differences were detected across ages in WTs for either SE or EE cohorts, EE-B KOs had significantly decreased latencies compared to environmental enrichment from adulthood (EE-A) KOs (**p = 0.003). **(B)** Velocity. Velocity was consistently and significantly faster in all WT groups compared to all KO cohorts (*p* < 0.008; not marked). EE did not impact velocity in Ten-m3 KOs at any age. A significant increase in velocity was seen in the EE WTs enriched in adulthood compared to standard-controls (*p = 0.013). Velocities of WTs enriched in adulthood were also greater than WTs enriched from weaning (gray *p = 0.018) and from birth (gray **p = 0.007). *p < 0.05; **p < 0.01.

**Figure 2:**
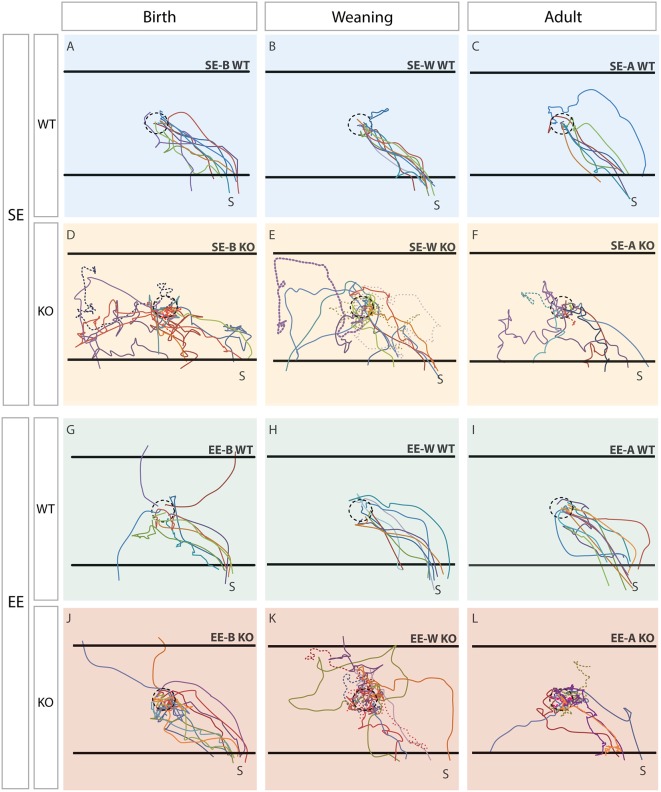
Trajectories reveal clear differences between genotypes which are corrected in Ten-m3 KOs following enrichment from birth. Traces show paths taken by individual mice. The trajectory of each mouse within a group is shown in a different color. The location of the dish containing the sunflower seed (starting point; see “Materials and Methods” section) is shown by the dotted circle in the center. The black horizontal lines at the “top” and “bottom” of each panel show pre-defined escape areas (see “Materials and Methods” section). Figures are oriented such that the shelter (S) is located in the bottom right corner. Each column represents a different age-group (birth, weaning, adult). The top row **(A–C)** illustrates trajectories of the standard-housed (SE) WT mice in each age group. All SE WTs displayed clear and efficient escape trajectories towards the shelter. The second row **(D–F)** shows SE KO mice. All SE KOs behaved very differently to WTs, tending to explore the cage extensively after the presentation of the stimulus. The trajectories of mice which did not reach an escape area within the time limit are shown by dotted lines. The third row **(G-I)** shows the trajectories of the WTs which experienced EE at some stage. These mice generally moved similarly to standard-housed WT mice, although three of the group which experienced EE from birth (EE-B WTs; **G**) fled initially to the “top” and “bottom” escape areas of the arena, rather than to the shelter directly. The bottom row **(J–L)** shows the trajectories of the EE KO mice. The KOs enriched from weaning or adulthood tended to exhibit random trajectories, similar to the standard environment control for weaning (SE-W) and adult group (SE-A) KOs. The EE-A KOs tended to hover around the center rather than heading to the exit. The trajectories of EE-B KOs **(J)**, however, appear much more similar to the paths taken by EE-B WTs than to those exhibited by standard environment control for birth group (SE-B) KOs, suggesting a recovery of visually-mediated behavior following EE from birth in Ten-m3 KOs.

In order to characterize the subject’s response to stimulus presentation, three specific parameters of each trajectory were recorded for further analyses: escape latency, escape velocity, and path efficiency. A different response to the stimulus, freezing (defined as remaining completely motionless for three or more seconds, with no visible movement of head, tail or limbs), was exhibited by one mouse (a SE-B KO); this subject was omitted from further behavioral analysis.

Latency was defined as the time taken by the subject to reach one of the escape areas (or end of stimulus presentation for those that did not escape) from stimulus onset. Mean velocity was calculated by dividing distance traveled during stimulus presentation by the escape latency. In order to obtain a better appreciation of flight trajectories, we developed an index which more accurately and sensitively reflected the escape response of all mice which we have termed path efficiency. For this, we divided the total distance traveled from stimulus onset to trial end (either when the subject reached an escape area or the end of stimulus presentation), by the straight line distance separating the starting and end-points of each mouse. A score of 1, therefore, indicated the mouse took the most efficient trajectory possible; progressively higher scores meant subjects took less efficient paths. Finally, “instantaneous” frame-by-frame velocity (sampled every 0.167 s) beginning 20 s prior to the onset of the stimulus, and ending either upon flight to an escape area (shaded region or shelter) or 20 s after the stimulus initiation, was plotted for each mouse as a heatmap using a custom script (MATLAB, Mathworks, MA, USA). Individual instantaneous velocity values were then averaged across all subjects for each group. The number of stimulus iterations prior to each mouse initiating flight (exiting the center area) was also assessed as a measure of the sensory processing/integration time before escape behavior commenced.

Measurements were analyzed using a three-way analysis of variance (ANOVA) with genotype, age and housing condition as factors. Differences between groups of interest were determined *via* pairwise testing, corrected for multiple comparisons (Bonferroni). Statistical analyses were performed using SPSS (IBM, NY, USA). A significance value of *α* = 0.05 was assumed for all statistical tests.

## Results

### Standard-Housed Ten-m3 KOs Show an Increased Response Latency Following Exposure to the Looming Stimulus

We first confirmed that SE adult (SE-A) WT mice respond reliably to the looming stimulus in our hands. Mice actively explored the chamber prior to stimulus onset. On presentation of a rapidly expanding disc overhead, all SE-A WTs displayed a clear flight response, typically initiating a rapid escape from their starting location towards the shelter in less than 250 ms. Mean escape latency (±standard error of the mean, SEM; defined as the time from initiation of the stimulus until the mice reached an escape zone) for SE-A WTs was 1.65 ± 0.48 s (*n* = 9; Figure 1A, right).

We then examined the response of the SE-A KO group. Unlike WTs, SE-A KOs did not exhibit reliable flight-like behavior. Instead, they typically moved quite slowly, with highly variable trajectories, many appearing to continue to explore the arena following stimulus presentation. Three of the ten SE-A KO mice tested did not reach an escape area within the maximum allotted time. Most notably, the mean escape latency for the SE-A KO group (8.85 ± 2.15 s, *n* = 10; Figure 1A, right) was over 4-fold longer than for WTs from this condition.

### Enrichment From Birth, but Not From Weaning or During Adulthood Reduces Escape Latency Following Exposure to the Looming Stimulus

The capacity of EE during adulthood (EE-A) to restore responsiveness to the looming stimulus in Ten-m3 KO mice was assessed. No obvious improvement in performance was observed. Four out of the 10 EE-A KO mice tested did not reach the escape area in the time considered. Indeed, mean escape latency (11.84 ± 2.18 s, *n* = 10; Figure 1A, right) for this cohort was actually slightly longer than in SE-A KOs. Thus, there was no evidence that enrichment during adulthood was able to drive a behavioral recovery in Ten-m3 KOs. For WT mice, EE during adulthood resulted in a slightly reduced mean response time (EE-A WT: 1.16 ± 0.29 s; *n* = 11) compared to standard housed animals (Figure 1A, right), the opposite of what was observed for KOs.

We asked whether EE might be more beneficial if it was provided during earlier development stages when neural circuits are forming and exhibit heightened levels of plasticity (Godement et al., 1984; Gordon and Stryker, 1996; Jaubert-Miazza et al., 2005). We first investigated the impact of 6 weeks of EE from birth (EE-B) on Ten-m3 KOs and WTs compared to age-matched standard-housed (SE-B) mice of both genotypes. As with the adult WT groups, the SE-B WT mice fled rapidly and reliably to an escape zone [latency (mean + sem): 1.85 ± 0.48 s, *n* = 10; Figure 1A, left]. Also consistent with observations of the adult cohort, SE-B KO mice displayed highly variable responses, with the mean latency over five-fold greater than that of WTs (11.61 ± 1.95 s, *n* = 10; Figure 1A, left). The EE-B KO mice, however, displayed much more rapid and reliable responses than the SE-B KOs. All EE-B KO mice fled to an escape area within the allotted time. The mean latency (5.52 ± 0.57 s, *n* = 12; Figure 1A, left) was less than half that of SE-B KOs. EE-B WTs, on the other hand, exhibited little change in escape latencies compared to SE-B WTs (EE-B WTs; 1.87 ± 0.42 s, *n* = 10; Figure 1A, left).

The duration of EE used in the birth group encompasses the entire postnatal developmental period of the mouse visual system, from axon ingrowth onto target structures (Godement et al., 1984; Jaubert-Miazza et al., 2005) all the way through to the critical period for monocular deprivation (Gordon and Stryker, 1996). Since our group has found that the anatomical correction of mismapped retinogeniculate projections in Ten-m3 KO mice requires exposure to EE between birth and weaning (Eggins et al., 2019), we investigated whether the EE-induced behavioral improvements showed the same temporal sensitivity. Therefore, we looked at the impact of EE commenced at weaning on response to the looming stimulus.

As observed in the other two age groups, enriched from weaning (EE-W) and standard-housed control (SE-W) WTs showed a robust and consistent response to the stimulus [SE-W WTs (mean ± SEM): 1.58 ± 0.46 s, *n* = 10; EE-W WTs: 1.51 ± 0.35 s, *n* = 9; Figure 1A, middle]. This contrasted markedly with mean flight latencies obtained for SE-W KOs (9.716 ± 2.19 s, *n* = 11; Figure 1A, middle). Unlike the KOs enriched from birth, the EE-W KO mice showed no evidence of reduced latency to escape compared to the SE-W KO cohort (EE-W KOs: 9.37 ± 1.74 s, *n* = 9; Figure 1A, middle), suggesting that enrichment from weaning is too late to rescue the flight response.

Statistical analysis revealed a highly significant effect of genotype (*F*_(1,109)_ = 194.727, *p* < 0.001), as well as a significant interaction between genotype, housing condition and age (*F*_(2,109)_ = 3.146, *p* = 0.047) on escape latency. Pairwise comparisons confirmed that the decrease in latency between SE-B KOs and EE-B KOs was significant (*p* = 0.001). Further, while there was a significant effect of genotype for SE mice from all age groups (*p* < 0.001), as well as within the EE-W and EE-A groups (*p* < 0.001), the latencies of EE-B KOs were not significantly different from EE-B WTs (*p* = 0.053). When compared across ages, EE-B KOs exhibited significantly decreased latencies compared to EE-A KOs (*p* = 0.003). Together, these results suggest that 6 weeks of EE can have a significant impact on flight behavior in Ten-m3 KOs with respect to latency, but this effect is highly dependent on the age at which EE is commenced.

### The Improved Performance of KOs Enriched Form Birth Is Not Due to Increased Velocity

While vision is clearly important for being able to detect the looming stimulus, the reduced latency of standard-housed KOs could also be due to other deficits which may also be ameliorated by EE from birth. We have previously shown that although quite mobile (Leamey et al., 2007), Ten-m3 KOs exhibit subtle deficits in motor learning (Tran et al., 2015) as well as kyphosis (Leamey et al., 2007). The increased latency to escape observed in KOs could, therefore, be a result of reduced motor ability.

Mean escape velocity following presentation of the looming stimulus was markedly reduced in standard-housed KOs (SE-B KOs: 7.36 ± 0.83 cms^−1^, *n* = 10; Figure 1B, left; SE-W KOs: 9.11 ± 1.40 cms^−1^, *n* = 11; Figure 1B, middle; SE-A KOs 6.94 ± 1.57 cms^−1^, *n* = 10; Figure 1B, right) compared to standard-housed WTs for all age groups (SE-B WTs: 21.44 ± 3.72 cms^−1^, *n* = 10; Figure 1B, left, SE-W WTs: 26.52 ± 4.31 cms^−1^, *n* = 10; Figure 1B, middle; SE-A WTs 25.86 ± 5.66 cms^−1^, *n* = 9; Figure 1B, right). EE did not increase mean velocity in KOs (EE-B KOs: 8.80 ± 0.89 cms^−1^, *n* = 12; Figure 1B, left; EE-W KOs: 8.17 ± 1.10 cms^−1^, *n* = 9; Figure 1B, middle; EE-A KOs: 6.72 ± 1.90 cms^−1^, *n* = 10; Figure 1B, right), regardless of the stage at which it was administered. Similarly, EE did not increase velocity for WTs enriched from birth (EE-B WT: 23.11 ± 4.54 cms^−1^, *n* = 10; Figure 1B, left) or weaning (EE-W WT: 24.35 ± 3.59 cms^−1^, *n* = 9; Figure 1B, middle). A slight increase following EE was observed in WTs enriched as adults (EE-A WTs: 38.78 ± 6.88 cms^−1^, *n* = 11; Figure 1B, right) compared to their standard-housed counterparts.

Statistical testing showed a highly significant effect of genotype on velocity (Figure 1B, *F*_(1,109)_ = 81.486, *p* < 0.001). No other effect of age, housing, or any significant interaction between genotype, housing condition, and age was observed. Pairwise comparisons revealed that KOs exhibited significantly decreased values across all ages and housing conditions compared to equivalent WTs (*p* < 0.008). Curiously, EE-A WTs displayed significantly greater velocities than SE-A WTs (*p* = 0.013), EE-B WTs (*p* = 0.007) and EE-W WTs (*p* = 0.018), suggesting a detectable effect of EE on the escape response for this group. The overall reduction in flight velocities in all KO groups compared to all WTs could, therefore, partially account for the increased escape latencies detected in Ten-m3 KO mice. Importantly, however, this does not explain the response recovery observed specifically in KOs enriched from birth. The age-dependent effect of EE on this particular group must, at least in part, be due to other factors.

### An Altered Number of Stimulus Iterations Prior to Exiting Arena Center May Contribute to Latency Differences

Velocity and escape latency described above were measured over the entire period from stimulus initiation to when subjects reached an escape zone (or the maximal time allowed). To better determine whether the changes in flight behavior is due to EE-induced improvements in sensory processing or integration, we examined how many iterations of the stimulus commenced prior to mice exiting the center zone. All standard-housed WT groups had mean (+SEM) stimulus iterations prior to escape approaching 1 (SE-B WTs:1.7 + 0.4; SE-W WTs: 1.4 ± 0.27; 1.78 + 0.46) suggesting an immediate response. This was not appreciably affected by EE (EE-B WTs 1.7 + 0.3; EE-W WTs 1.22 ± 0.15; EE-A WTs 1.45 + 0.28). Substantially more stimulus iterations were presented to all KO groups before they initiated movement away from the center (SE-B KOs: 6.6 + 1.33; SE-W KOs: 6.4 + 1.57; SE-A KOs: 9.0 + 1.91). Importantly, EE-B KO mice showed evidence of a decrease in the number of stimulus iterations before commencing their escape (3.8 + 0.46) compared to the SE-B KOs, although these values did not reach WT levels. EE did not reduce the number of iterations prior to exiting the center in EE-W KOs (6.89 + 0.86) and an increased value was found for EE-A KOs (11.0 + 1.71).

A univariate ANOVA with housing, genotype, and age as independent factors showed that differences were present in the number stimulus iterations to response, with significant effects for genotype (*F*_(1,109)_ = 93.114, *p* < 0.001) and age (*F*_(2,109)_ = 5.795, *p* = 0.004), as well as an interaction between these two (*F*_(2,109)_ = 5.782, *p* = 0.004). Pairwise comparisons revealed that SE-B vs. EE-B KOs exhibited a trend towards a difference (*p* = 0.050); no other differences were observed between SE and EE groups across genotype and age (*p* > 0.170). Pairwise comparisons revealed that the number of iterations commenced before response initiation was different between WTs and KOs for all housing and age groups (*p* ≤ 0.001), except those enriched from birth (EE-B KOs vs. EE-B WTs, *p* = 0.129). Across ages, EE-A KOs values were different from EE-W KOs (*p* = 0.012) and EE-B KOs (*p* < 0.001); no other age-related changes were detected (*p* ≥ 0.107). Together these findings suggest that enrichment from birth affected the time required for Ten-m3 KO mice to initiate a response to the looming stimulus, consistent with a partial recovery of visual function in this cohort.

### Enrichment From Birth Improves the Path Efficiencies of Escape Trajectories in Ten-m3 KO Mice

The flight response assesses whether subjects avoid a perceived descending aerial threat by moving away from a likely point of impact. Critical to this idea is that mice would target potential areas of safety as rapidly and directly as possible. Escape latency, stimulus iterations prior to escape commencement, and velocity provide very little information regarding how and where subjects flee to escape the looming stimulus. In order to gain further insight into how EE was impacting flight behavior specifically, we examined the escape trajectories of both WT and KO mice.

Qualitative assessment revealed marked differences in the flight paths of all SE WT compared to all SE KO groups. SE WTs from all age groups took consistent and quite direct routes from their starting location towards the shelter (Figure 2, top row). This contrasted with the trajectories of all SE KOs which were much more variable and circuitous (Figure 2, 2nd row). Multiple mice in each age group failed to reach an escape area within the time allowed (trajectories of these mice are shown as dotted lines in Figure 2). EE did not produce any marked changes in WT trajectories (3rd row), with the minor exception that some EE-B WTs fled initially to either the “top” or “bottom” escape zones within the arena (see Figure 2 and “Materials and Methods” section for details), rather than directly towards the shelter.

EE from birth in Ten-m3 KOs produced a marked change in escape routes (Figure 2, bottom row, left), compared to all other KO groups. EE-B KOs took much more consistent and direct paths to the shelter. Similar to EE-B WTs, a subset fled initially towards the “top” escape region within the arena. Notably, mice within this group, but no other KO group, all reached an escape area within the allotted time. EE from weaning or in adulthood (Figure 2, bottom row middle and right respectively) did not noticeably improve the trajectories of Ten-m3 KO mice compared to standard-housed controls, although a tendency for some EE-A KOs to hover near the center of the arena could be observed.

In order to quantitatively examine flight paths, we generated an index that assessed the degree to which an individual’s escape trajectory deviated from an ideal minimum-deemed “path efficiency” (a score of 1 indicates the most efficient trajectory possible; see “Materials and Methods” section). Unlike escape latency and velocity, this metric is less dependent on subtle differences in motor function.

**Figure 3:**
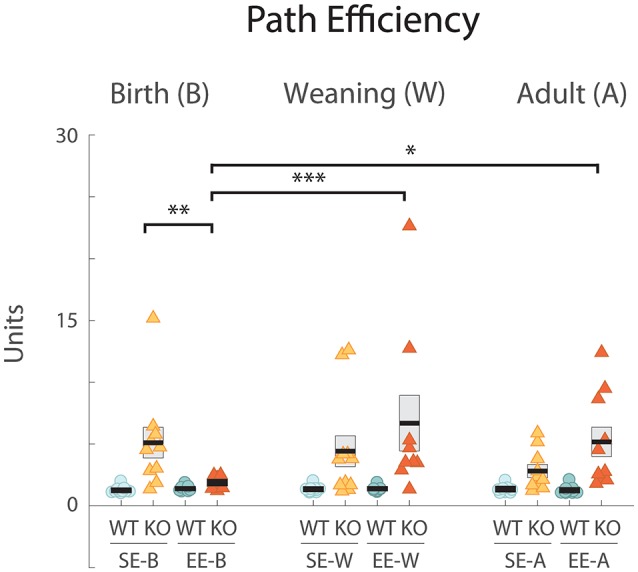
EE from birth improves the path efficiency index in Ten-m3 KOs. Graph plots path efficiency (see “Materials and Methods” section). Conventions as for Figure 1. All WTs scored efficiency values which clustered slightly above the ideal value of 1. All standard-housed KO groups had higher and more variable indices indicating less efficient trajectories. All SE KO groups were significantly different from all SE WT cohorts (*p* < 0.001; not marked). EE-B KOs exhibited significantly lower (improved) path efficiencies compared to SE-B KOs (**p = 0.007). Further, efficiencies in the former group were significantly different from groups enriched from weaning (EE-W KOs; ***p < 0.001) and as adults (EE-A KOs; *p = 0.019). A tendency towards higher efficiency scores (less efficient pathways) in KOs enriched as adults compared to standard-housed controls was detected but did not reach significance (EE-A KOs vs. SE-A KOs *p* = 0.059). When comparing between genotypes, KOs enriched from birth were not detectably different from WTs enriched from birth (*p* = 0.687). Values for KOs enriched from weaning and in adulthood, however, were very different from their respective WT groups (*p* < 0.001; *p* = 0.002; not marked). *p < 0.05; **p < 0.01; ***p < 0.001.

All standard-housed control (SE-B WT: 1.26 ± 0.09, *n* = 10; Figure 3, left; SE-W WT: 1.35 ± 0.09, *n* = 10; Figure 3, middle; SE-A WT: 1.35 ± 0.11, *n* = 9; Figure 3, right) and corresponding enriched (EE-B WT: 1.40 ± 0.07, *n* = 10, EE-W WT: 1.39 ± 0.08, *n* = 9; EE-A WT: 1.27 ± 0.10, *n* = 11) WTs scored similarly for path efficiency with scores approaching the ideal value of 1. Standard-housed Ten-m3 KO scores, on the other hand, were generally higher (i.e., exhibited less efficient paths) and showed a greater degree of variability (SE-B KO: 5.106 ± 1.24, *n* = 10; Figure 3, left; SE-W KO: 4.42 ± 1.24, *n* = 11; Figure 3, middle; SE-A KO: 2.81 ± 0.52, *n* = 10; Figure 3, right).

EE did have a beneficial impact, but only in KOs that were enriched from birth. EE-B KOs had path efficiency values that were similar to those of WTs (EE-B KO: 1.88 ± 0.12, *n* = 12; Figure 3, left). Ten-m3 KO mice that experienced EE at later stages exhibited values that were no different from standard-housed KOs (EE-W KO: 6.69 ± 2.27, *n* = 9; Figure 3, middle; EE-A KO: 5.17 ± 1.19, *n* = 10; Figure 3, right). Importantly, the pattern of EE-mediated improvement was similar to that seen for latency, suggesting that improved path efficiency at least partially accounts for performance differences detected in the EE-B KOs.

Statistical analysis revealed that genotype had a highly significant effect on path efficiency (*F*_(1,109)_ = 35.548, *p* < 0.001). A significant interaction between genotype, housing condition, and age was also detected (*F*_(2,109)_ = 3.626, *p* = 0.030). Pairwise comparisons confirmed that enrichment from birth led to more efficient escape trajectories in KOs, with path-efficiency scores significantly decreased (indicating more efficient trajectories) in EE-B KOs compared to SE-B KOs (*p* = 0.007; Figure 3, left). Further, EE-B KO values were no different from those of the corresponding WTs (*p* = 0.687), while scores for EE-W KOs and EE-A KOs, were very different from their corresponding WT groups (*p* < 0.001; *p* = 0.002, respectively; not shown). Consistent with this, EE-B KOs also exhibited significantly lower path efficiency scores compared to both EE-W KOs (*p* < 0.001) and EE-A KOs (*p* = 0.019). A trend towards higher efficiency scores (i.e., less efficient paths) was observed in EE-A KOs compared to standard-housed controls, but this did not reach significance (*p* = 0.059). These findings strongly support the qualitative analysis of trajectories and underscore an age-dependent impact of EE on the recovery of visually-mediated behavior in Ten-m3 KOs.

### Behavioral Differences Are Not Apparent in the Period Immediately Prior to Stimulus Presentation

Our data indicate that mice exhibit differential escape responses to a visually-presented looming stimulus across genotype, housing, and (enrichment initiation) age. It is, however, possible that the changes in flight behavior, particularly in terms of escape efficiency, observed in SE, EE-W, as well as EE-A Ten-m3 KOs, could be due to varying familiarity with escape zone locations (including the shelter) within the test arena. If this were the case, those cohorts that exhibited deficits in-flight responses may have had little or no visitations of the shelter area as a group, before stimulus onset. When we examined general exploratory behavior 2 min prior to stimulus presentation, we found that the vast majority of mice (93%) across all groups visited the shelter at least once during this period (a range of 80–100% across groups). These values showed no obvious relationship with post-stimulus behavior.

**Figure 4:**
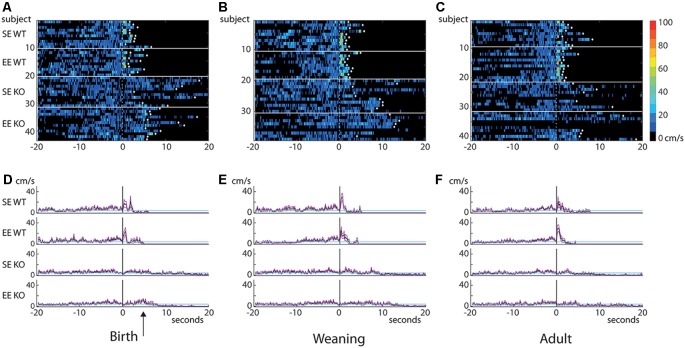
Behavioral differences do not emerge until the post-stimulus interval. Heatmaps of “instantaneous” velocity calculated frame-by-frame for the 20 s prior to and after the initiation of the visual stimulus **(A–C)**. The stimulus was presented at 0 s (dotted line). Each row in the heatmap represents an individual mouse (housing and genotype groups indicated on the left). Heatmaps are thresholded to show only movement of ≥4 cm/s (velocities less than 4 cm/s are depicted in black). White diamonds indicate that a given mouse reached an escape zone. Graphs plotting mean (cm/s; solid black traces) and standard errors of the mean (magenta lines) of instantaneous velocity across time for each group are shown in **(D–F)**. Cyan lines indicate 4 cm/s threshold. Data from the birth group are shown on the left **(A,D)**, weaning in the center **(B,E)** and adult on the right **(C,F)**. There are no apparent differences in pre-stimulus velocity between groups, suggesting that all cohorts explored the environment to a similar degree during this period. Dramatic differences emerge in the immediate post-stimulus interval where all WT groups show a brief but marked increase in velocity. KO mice do not show this increase. Only a small, slightly delayed post-stimulus increase can be seen in the EE-B KOs (**D**, arrow). Note that all WTs escaped well within the allowed time, but the only KO group where all members reached an escape zone was the EE-B KOs (escape denoted by white diamonds).

Moreover, an examination of “instantaneous” (frame-by-frame) velocity 20 s before and after stimulus presentation (depicted as heatmaps; Figures 4A–C) showed no obvious qualitative differences in movement profiles during the pre-stimulus period, despite the dramatic and varied responses (brief, rapid peaks just after stimulus presentation particularly for WTs compared to the much flatter responses observed in KOs, consistent with analyses described earlier; see averaged values for groups; Figures 4D–F) emerging in the post-stimulus interval. Curiously, EE-B KOs on average showed a slight post-stimulus increase in velocity compared to SE-B KOs, but this was much smaller and delayed compared to all WT groups (arrow; Figure 4D).

Thus, despite the observed variability in visually-driven responses, the lack of a comparable difference in pre-stimulus behavior suggests that familiarity with the surroundings is not a major factor in the differing performance levels observed between the groups assessed. Taken together, the data supports the suggestion that the primary remediation in the EE-B KO group is not due to an impact on motor performance, but rather due to the selection of a more efficient escape trajectory following the presentation of the stimulus.

## Discussion

This study has revealed that standard-housed Ten-m3 KO mice exhibit a deficient flight response to a potentially threatening visual stimulus presented to their dorsal (binocular) visual field. Further, EE from birth, but not from weaning or during adulthood, is able to rescue this visually-mediated escape behavior. This response recovery correlates well with the previously reported selective reduction of the most aberrantly targeted ipsilateral retinogeniculate projections (Eggins et al., 2019), exclusively in KOs enriched from birth. To our knowledge, this is the first study to demonstrate that EE can partially rescue a functional deficit associated with genetically-determined axonal wiring defect, but only when administered during an early “critical period” of sensitivity to this intervention.

### Technical Limitations

This study has used the analysis of a single visually-mediated behavior to infer possible improvements in the function of a miswired circuit. This particular test was chosen as it specifically assesses dorsal (binocular) visual field function and does not require learning, an attribute that may be compromised in Ten-m3 KOs (see below). While the data presented are consistent with the possibility that binocular visual function is improved in these mice, they are not definitive. Importantly, experiments that assess other visual properties known to be affected by enrichment such as acuity (Prusky et al., 2000; Cancedda et al., 2004) should be conducted to reveal if, and to what degree, they contribute to the changes described here.

The term binocular vision is used here to indicate that the subject had both eyes open and was responding to a stimulus displayed to the region of binocular overlap above the animal. While binocularity imparts a number of specific visual functions such as depth perception, the degree to which this, or any other characteristic derived from the integration of input from both eyes contributes to the changes observed here is not known. Further work will be required to address which aspect of binocular vision is most critical for the enrichment dependent improvements in-flight responses observed in Ten-m3 KO mice.

Finally, since this is a behavioral study in freely moving animals, it was not possible to accurately control for head or body position. While the use of a sunflower seed helped to standardize animal position relative to the stimulus launch point overhead, some minor variation in the region of the visual field from which the stimulus initiated expansion was inevitable. Although this could potentially underlie some of the individual response variability observed within groups, it does not explain the dramatic differences in the flight responses that were observed between some cohorts.

### EE From Birth Induces the Recovery of an Ethologically-Appropriate Visually-Driven Behavior in Ten-m3 KOs

Consistent with previous work (Yilmaz and Meister, 2013), WT standard-housed mice exhibited a robust and reliable flight response to an expanding, looming disk, presented above the animal, emulating an aerial predator. In contrast, standard-housed Ten-m3 KO mice did not respond reliably or efficiently, consistent with visual deficits previously reported in these mice (Leamey et al., 2007; Merlin et al., 2013). Exposure to an enriched environment for 6 weeks from birth was enough to induce a significant behavioral recovery in these KOs. This could not be easily explained by improvements in motor function or ambulation, as mean velocities over distances traveled post-stimulus did not increase with enrichment. Further, no differences in pre-stimulus velocities were apparent between groups. Rather, EE from birth was found to improve the path efficiency of flight trajectories taken by the Ten-m3 deficient mice following initiation of the stimulus.

The observation that KOs enriched from birth took more direct routes to escape a perceived potential aerial predator than any other KO group suggests that they may have gained an increased awareness of their surroundings. This includes the presence of the stimulus—which can only be detected visually—as well as other aspects of the arena such as overall layout and the location of escape areas. The looming stimulus was presented to the dorsal visual field; the region whose central representation is most severely disrupted in Ten-m3 KOs. The temporal sensitivity of the behavioral recovery to EE correlates very well with that which we previously observed for miswired ipsilateral projections, and corresponds to the same region of the visual field (Eggins et al., 2019). It is, therefore, very tempting to speculate that there may be a direct link between this wiring correction and behavioral recovery. While a relationship is likely, other possible factors should be considered. Notably, Ten-m3 is expressed in neural circuits other than the retinogeniculate pathway (Zhou et al., 2003; Leamey et al., 2007, 2008; Dharmaratne et al., 2012; Tran et al., 2015; Berns et al., 2018), so the function of these networks may also be compromised in KOs. Further, these circuits may also have been impacted by EE, contributing to the observed behavioral recovery.

The response to the looming stimulus is considered innate (Yilmaz and Meister, 2013), and was chosen here to minimize any potential effects of altered learning in Ten-m3 KOs. Nevertheless, the flight response can be affected by changes in spatial acquisition (Vale et al., 2017). The hippocampus is well-known for its important roles in spatial learning and navigation (reviewed in Moser et al., 2017; Rolls, 2018). Ten-m3 is highly expressed in the hippocampus (Zhou et al., 2003; Leamey et al., 2008), and hippocampal connectivity is impacted in standard-housed Ten-m3 KOs (Berns et al., 2018; Leamey and Sawatari, 2019). While there were no apparent differences in the exploration of the arena in the pre-stimulus interval, or awareness of the presence of the shelter, we cannot rule out the possibility that defects in spatial navigation could have influenced the performance of the standard-housed Ten-m3 KO mice. A beneficial effect of EE on hippocampal function could, therefore, have contributed to the behavioral recovery in Ten-m3 KOs. The early, narrow time window of sensitivity to EE seen here does not fit well with hippocampal changes being the primary driver, however, as there is strong evidence that exposure to EE from weaning (Bernstein, 1973; Kempermann et al., 1997; van Praag et al., 2000) and even into old age (Speisman et al., 2013; Neidl et al., 2016; Cortese et al., 2018) can have a beneficial impact on spatially-dependent hippocampal function.

It is also possible that in addition to their visual deficits, Ten-m3 KOs may lack the motivation to escape from the stimulus. This is broadly consistent with changes in the thalamostriatal pathway in Ten-m3 KOs (Tran et al., 2015). If this is the case, then enrichment from birth, but not at later stages, appears to restore this motivation. Further work is required to assess changes in motivation in Ten-m3 KO mice under standard and enriched conditions.

Visually-driven flight behavior requires activation of the superior colliculus (SC) as well as the amygdala (Wei et al., 2015). While Ten-m3 does not appear to be present in the amygdala (CAL, unpublished observations and Allen Brain Atlas), there is prominent expression of the axon guidance protein in the SC (Dharmaratne et al., 2012). Ipsilateral retinal projections targeting the SC also show a topographical mismapping in standard-housed Ten-m3 KOs (Dharmaratne et al., 2012). Accordingly, it is possible that the behavioral effects we have observed here are also due to an impact of EE from birth on mismapped ipsilateral retinocollicular terminals in Ten-m3 KOs.

EE may also induce changes at other levels of the visual pathway, including the retina (Landi et al., 2007) and V1 (Ciucci et al., 2007; Sale et al., 2007; Levine et al., 2017), which may contribute to the behavioral recovery. In V1, exposure to EE has been shown to accelerate, in juvenile rodents (Cancedda et al., 2004; Baroncelli et al., 2016), as well as re-activate, in adult (Sale et al., 2007; Baroncelli et al., 2010) and even-aged rodents (Scali et al., 2012; Greifzu et al., 2016), the capacity for ocular dominance plasticity. EE may be exerting a similar effect in V1 in our mice which could be contributing to the observed recovery improvement in flight responses. Importantly, however, while just 2–3 weeks of EE is able to induce ocular dominance plasticity in V1, and drive recovery from amblyopia well into adulthood (Sale et al., 2007; Baroncelli et al., 2010), 6 weeks of EE was unable to induce a behavioral recovery in Ten-m3 KO mice if it was initiated at 3 weeks of age or older. Thus, while EE applied at later stages may enhance cortical plasticity in Ten-m3 KOs, this is insufficient to drive the improved responses described here. Our data suggest that EE from birth induces additional or distinct mechanisms that may work in concert with EE-derived benefits in V1 (and/or on other pathways) to drive behavioral recovery.

While the potential influence of EE on these and other circuits is acknowledged and may well contribute, we propose that EE from birth induced correction of retinogeniculate mapping likely plays a pivotal role in the recovery of flight behavior seen here, by driving changes in cortical activation and output. V1 of standard-housed Ten-m3 KOs exhibits suppression of responsiveness to binocularly presented stimuli (Merlin et al., 2013). This is in large part due to the mismapped input from the two eyes; monocular inactivation leads to a recovery of visual function (Leamey et al., 2007; Merlin et al., 2013). Since the primary ipsilateral retinogeniculate mismapping is significantly corrected in Ten-m3 KOs enriched from birth (Eggins et al., 2019), it is likely that cortical visual responsiveness is also restored to some degree. Enhanced EE-induced OD plasticity in V1 may well contribute to this. The cortex, in turn, provides strong input to the SC (Olavarria et al., 1982; Olavarria and Montero, 1989; Wang and Burkhalter, 2013; Zingg et al., 2017). Activation of corticofugal projections has been shown to trigger defensive responses in mice (Zingg et al., 2017), and to regulate the responsiveness of collicular neurons to the looming stimulus (Zhao et al., 2014). Thus, the induced changes in retinogeniculate mapping in Ten-m3 KOs due to EE from birth may revive cortical modulatory drive of SC circuits, suggesting they could make a direct and vital contribution to induce the recovery of circuit function critical for the flight response. Further studies that reveal the impact of EE on retinocollicular patterning, as well as a more direct evaluation of V1 responsiveness in Ten-m3 KO mice will be required to determine the exact role each of these pathways plays in driving this stereotyped, visually evoked escape behavior.

The absence of any behavioral rescue in the enriched adult and weaning KO groups correlates with a lack of observable change in the degree of the mismapping present in their retinogeniculate pathways (Eggins et al., 2019), suggesting a link between these events. Although correlating well with our anatomical results, the absence of an effect of EE in the weaning group was somewhat surprising, as the time during which enrichment was administered overlapped with periods of refinement and high plasticity in the dLGN (Hong et al., 2014), as well as the visual cortex (Gordon and Stryker, 1996). Our data suggest that the pre-weaning period may be “critical” for EE to enable functional visual recovery from the profound deficits induced by the miswiring of projections in Ten-m3 KOs. This confined temporal window suggests that the regulatory mechanisms may be distinct from those that underlie recovery from monocular deprivation in V1, where sensitivity to EE lasts throughout life (Sale et al., 2007; Baroncelli et al., 2010; Scali et al., 2012; Greifzu et al., 2016).

### Differences With Other EE Protocols

The impact of EE during the pre-weaning period is thought to be derived from effects on maternal care, which has been shown to be enhanced by EE (Cancedda et al., 2004; Begenisic et al., 2015; reviewed in Sale et al., 2014). Since EE during the pre-weaning period is critical for the effects we observed here, it is likely that it is at least partially mediated by maternal effects. Despite the fact that many benefits of post-weaning EE have been reported (e.g., Rosenzweig et al., 1964; Bernstein, 1973; van Dellen et al., 2000; reviewed in Sale et al., 2014), no beneficial impact of EE from weaning or later was observed here on the response of Ten-m3 KO mice to the looming stimulus. It is likely that EE may have exerted other effects which were not detected by our analysis. Ten-m3 KOs enriched as adults did show a trend towards a change in path efficiency compared to standard-housed controls, but in a manner that indicated they were less responsive to the stimulus. This could be due to an enrichment-induced reduction in anxiety levels (Benaroya-Milshtein et al., 2004; Hüttenrauch et al., 2016), coupled with little improvement in the awareness of the stimulus due to the lack of an effect on miswired retinal projections (Eggins et al., 2019).

WT mice showed little change in their flight responses following enrichment at any stage. An exception was that WTs enriched as adults showed an increase in velocity compared to standard-housed controls. This could reflect the benefits of access to an exercise wheel on motor coordination and/or muscle strength in adulthood. The lack of a more substantial impact of EE on WT mice was somewhat surprising, given that beneficial effects of enrichment have been well-documented for these mice (reviewed in Sale et al., 2014). This may be due to a “ceiling” effect, as the relatively simple task used here minimally engages higher-order perceptual and executive pathways in a healthy WT mouse. More cognitively demanding behavioral tasks may be required to observe any detectable impact of EE in these animals (Rountree-Harrison et al., 2018).

### Path Efficiency Provides Useful Insights Regarding Flight Behavior

Previous analyses utilizing the looming stimulus have used escape latency as a primary measure of performance. This is likely appropriate for healthy WTs which exhibit a consistent, stereotyped flight response. For models of disorders or disease, however, where there are multiple potential defects that could affect sensory, cognitive and/or motor performance, parameters that account for how and where these animals escape the visual threat is required to fully characterize their behavior. The path efficiency index used here, which compares actual trajectories relative to an ideal escape route, seemed to best reflect the changes observed across different genotypes, housing, as well as age groups.

### Implications for the Timing of Therapies to Ameliorate Neurodevelopmental Disorders

Our work showing a clear age-dependent effect of EE on the rescue of behavioral function has obvious implications for the development of non-invasive therapies in humans. Enhanced sensory experience has been revealed to help ameliorate symptoms in autistic children (Woo and Leon, 2013; Woo et al., 2015; Aronoff et al., 2016). Interestingly, a greater impact of interventions commenced prior to (compared to after) the age of 2 years has been shown (MacDonald et al., 2014), correlating well with our findings.

## Conclusion

This work shows that EE is able to rescue an ethologically-relevant visually-mediated behavior in Ten-m3 KOs. The timing of exposure to EE was found to be critical, however, as the recovery was only seen in animals that were exposed to EE during early postnatal development: no effect was observed in animals that commenced EE from weaning or later. This correlates well with our previously reported enrichment induced rescue of miswired ipsilateral retinogeniculate inputs in these mice—which showed the same temporal sensitivity to EE—suggesting that the anatomical correction may be related to the observed functional improvements, although further work is needed to confirm this. Our study observed that, in addition to its previously-reported ability to enhance cortical plasticity at all stages of life, EE can also enable recovery of an innate behavioral response in mice where this is usually compromised. Importantly, this capacity is only present during an early critical period.

## Data Availability Statement

All datasets generated for this study are included in the article.

## Ethics Statement

This study was carried out in accordance with the recommendations of the Australian code for the care and use of animals for scientific purposes (Edition 8), National Health and Medical Research Council Guidelines, Animal Welfare Committee. The protocol was approved by Animal Ethics Committee of the University of Sydney.

## Author Contributions

CL and AS designed the study and wrote the article. JB, JP, and DB collected the data. JB, DB, CL, and AS analyzed the data.

## Conflict of Interest

The authors declare that the research was conducted in the absence of any commercial or financial relationships that could be construed as a potential conflict of interest.

## Abbreviations

EE: environmental enrichment
SE: standard laboratory housing environment
KO: knockout
dLGN: dorsal lateral geniculate nucleus
V1: primary visual cortex
WT: wildtype
EE-B: environmental enrichment from birth
EE-W: environmental enrichment from weaning
EE-A: environmental enrichment from adulthood
SE-B: standard environment control for birth group
SE-W: standard environment control for weaning group
SE-A: standard environment control for adult group
SEM: standard error of the mean
ANOVA: analysis of variance.
